# Supplementary material for: In vivo structure and dynamics of the SARS-CoV-2 RNA genome
Source: Nat Commun. 2021 Sep 28;12:5695. doi: 10.1038/s41467-021-25999-1 (PMC8478942; doi:10.1038/s41467-021-25999-1)

Supplementary Figure legends

Supplementary Fig.1| Heat maps of RNA-RNA interactions along the SARS-CoV-2 gRNA in particular replicates Similar to Fig. 1c, except that each plot represents a particular replicate. The bottom heatmaps show corresponding non-ligate controls.

Supplementary Fig.2| Correlation of chimeric read counts between samples Scatter plots show correlation of chimeric read counts between ligated samples (a and b), as well as ligated and non-ligated samples (c, d and e). r: two sided Pearson's correlation coefficients, P: Pearson's correlation test p-values in all the panels.

Supplementary Fig.3| proportion of 5′-3′and 3′-5′ chimeras in simplified SPLASH and COMRADES a, simplified SPLASH chimeras from indicated samples are divided into short-distance chimeras (distance < 200nt) and long-distance chimeras (distance > 200nt) according to the distance of the genome of the two segments. **b**, same as a, but chimeras are from COMRADES data.

Supplementary Fig.4| Structure of 5′-UTRs a, chimeras per million total mapped reads contact matrix in 5′-UTR region. Stem-loops were indicated as retangels. **b**, Canonical SARS-CoV-2 5′-UTR structure in C sample. Basepairing of indicated arches. Colors represent the log2 base pairing score normalized by total mapped read counts. **c**, Same as **b,** except that Colors represent the log2 base pairing score normalized by total mapped read counts in L samples. **d**, Same as **b,** except that Colors represent the log2 base pairing score normalized by total mapped read counts in V samples. **e**, ROC curves for simplified SPLASH data on 5'-UTR (using known consensus base-pairing information as true positives) compared to COMRADES data (Ziv O et al., 2020). **f,** Bar chart showing positive predictive value (PPV) and sensitivity in three indicated stages.

Supplementary Fig.5| Structure of 3′-UTRs a, chimeras per million total mapped reads contact matrix in 3'-UTR region. **b**, Canonical basepairing of SARS-CoV-2 3′-UTR. Colors represent the log2 base pairing score normalized by total

mapped read counts.. **c**, Alternative conformation B of SARS-CoV-2 3′-UTR. Colors represent the log2 base pairing score normalized by total mapped read counts. **d,** Alternative S2M structure of SARS-CoV-2 3′-UTR. Colors represent the log2 base pairing score normalized by total mapped read counts. **e,** same as b), but Colors represent the log2 base pairing score normalized by total mapped read counts from COMRADES data. **f,** same as c), but Colors represent the log2 base pairing score normalized by total mapped read counts from COMRADES data. **g,** same as d), but Colors represent the log2 base pairing score normalized by total mapped read counts from COMRADES data.

Supplementary Fig.6| Structure of genome cyclization a, chimeras per million total mapped reads contact matrix supporting genome cyclization. **b**, Base pairing 5′-UTR and 3′-UTR in C, L and V samples. log2 base pairing score normalized by total mapped read counts from indicated sample.

Supplementary Fig.7 characteristics of TRS-L chimeras a and **b**, Viewpoint histograms showing binding positions of the TRS-L region (first 100nt) along the SARS-CoV-2 genome in indicated samples for stringent (a) and relax pipeline(b). 3′-5′chimeras and 5′-3′ chimeras were separately plotted. As controls, we also plotted interaction position in non-ligated samples and in RNA-seq data. To show that the interaction peaks especially from 3′-5′ chimeras are specific. **c**, Schematic of ligation products and mapping details of chimeras. Junction sites are defined as indicated. **d**, Same as Fig.2C, but the chimeras are identified by relax pipeline. **e**, Same as Fig.2e, but the chimeras are identified by relax pipeline, which results in plenty of 5′-3′ chimeras, so 5′-3′ chimeras were also showed, indicating highly consensus of junction sites.

Supplementary Fig.8| mapping details of 3′-5′ chimeras supporting TRSL and TRS-B interactions a, similar to Fig.2e, lines show mapping of 3′-5′ chimeras overlapping the TRS-L: interactions. **b**, same to **a,** but chimeras are called from COMADES data (gRNA sample1, SRR12252274)

Supplementary Fig.9| base pairing of TRSL interactions similar to Fig.2f, all the base pairs are coloured by log2 base pairing scores normalized by total mapped reads in indicated samples.

Supplementary Fig.10| Identification and validation of novel sgRNAs a and **c**, Contact matrix of 3′-5 chimeric reads indicated specific interactions of TRS-L and 3.9K (a) and 12.3K (regions). Color depict chimeric reads per 1million mapped reads (CPM). **b** and **d**, Randomly selected chimeric reads showed distribution of reads aounrd junction sites. The redlines indicated 3′arm in chimeric reads, while blue lines indicated 5′arrm in chimeric reads. **c** and **e**, base pairing of TRS-L region and 3.9k (**c**) and 12.3K(**e**) regions **f** and **g**, Sanger sequencing validation of 3.9K (**f**) and 12.3K(**g**) novel sgRNAs from independent non-crosslinked samples. Bases around junction sites were shown.

Supplementary Fig.11| base pairing around FSE a, base pairing of canonical and alternative structures of FSE in indicated samples. Paired bases are coloured by log2 base pairing scores. **b**, base pairing of canonical and alternative structures of FSE in indicated samples from COMRADES data. Paired bases are coloured by log2 base pairing scores.

Supplementary Fig.12| base pairing of additional FSE arches base pairing of additional FSE arches of FSE in indicated samples. Paired bases are coloured by log2 base pairing scores.

Supplementary Fig.13| mapping details of chimeras spanning FSE arches lines show mapping of chimeras overlapping the TRS-L: interactions. blue lines show 5′-half of chimeras, and red lines show 3′-half of chimeras.

Supplementary Fig.14| mapping details of chimeras from COMRADES data spanning FSE arches same as Supplementary Fig.13, except that data are from COMRADES data.

Supplementary Fig.15| Clustering and PCA of samples a, cluster of Pearson correlation efficiencies between samples. **b**, Principal component analysis (PCA) interaction data from samples indicated in A.

Supplementary Fig.16| quantification of interactions in different phase of SARS-CoV-2 virus life cycle a, Arc plots show distribution of enriched interaction pairs in indicated samples. For clear visualization, continuous 10nt-bin pairs were merged. 5'-UTR-mediated interactions are marked in red, 3'-UTR-mediated interactions are marked in blue, and others are marked in gray. **b**, upset plot show overlapping of enriched interactions (10nt-bin pairs) in indicated samples. **c**, upset plot show overlapping of enriched interactions (10nt-bin pairs) in indicated samples, gRNA and mRNA interaction clusters from COMRADES data (Ziv O et.al, 2020) were also included. **d**, Histograms of spans of enriched interactions in each stage. **e**, RNA-interactions labelled by RNA coordinates.

Supplementary Fig.17| Dynamics of interactions in different phase of SARS-CoV-2 virus life cycle a, Vennplot overlap of differential interactions of VvsC and VvsL. **b**, correlation of log2FCs as indicated. **c**, Arc plots show strengthened and weakened interactions. Differential interactions with abs(log2FC) cutoff >= ±5 were plotted **d**, Heatmaps show log2FC of all the TRS-L (first 100nt) interactions in different comparisons. Note that almost all interactions are not changed or weakened, except that TRS-L: S interaction is strengthened.

Supplementary Fig.18| Characteristics of boundaries in different phase of SARS-CoV-2 virus life cycle a, Correlation of insulation score in different samples. r: two-sided Pearson’s correlation coefficient, P: Pearson’s correlation test p-value. **b**, Upset plot show overlaps of boundaries in different stages. C, L and V samples shared boundaries comprised most of boundaries. **c**, Histograms of domain length in different samples. **d**, Violin plot compare ratio of intra-domain/inter-domain interactions. Two-sided Wilcoxon rank sum tests were used to compare indicated groups, no adjustment of multiple comparisons was made. Boxplots show largest (upper whisker), smallest (lower whisker), 50% quantile (center), upper hinge (75% quantile) and lower hinge (25% quantile).

Supplementary Fig.19| Shannon entropy of SARS-CoV-2 genome a, Shannon entropy values along the SARS-CoV-2 genome. **b**, density plot of Shannon entropies from different samples. Shannon entropies were highest in L while lowest in V. C < L (one-sided “less” Wilcoxon rank sum test, P=1.61e-258, W=2175030), V<L (one-sided

“less” Wilcoxon rank sum test, P<2.2e-999, W=1622582), V<C (one-sided “less” Wilcoxon rank sum test, P =3.62e-51, W= 3463729). **c**, Dotplots show correlations between Shannon entropy values and Insulation scores. r: two-sided Pearson’s correlation coefficient, P: Pearson’s correlation test p-value.

Supplementary Fig.20| SARS-CoV-2 recombination with several SARS related coronaviruses. SARS-CoV-2 recombination with several SARS related coronaviruses. SimPlot genetic similarity plot between SARS-CoV-2 Wuhan-Hu-1 and representative CoV sequences using a 200–base pair (bp) window at a 10-bp step and the Kimura two-parameter model. Regions between breakpoints (99% confidence) were indicated as black lines on the top. Domain boundaries were indicated as breaks on x axis.

Supplementary data1

Statistics of mapping and chimeras of all the samples

Supplementary data2

contact matrix of each group of datasets.

Supplementary data3

DESeq2 statistics for enrichment of interaction bin pairs in ligated samples. The statistics of baseMean: mean of normalized counts for all samples; log2FoldChange: log2 fold change; lfcSE: standard error; stat: Wald statistic; pvalue: Wald test p-values; padj: Benjamini & Hochberg adjusted p values.

Supplementary Fig1

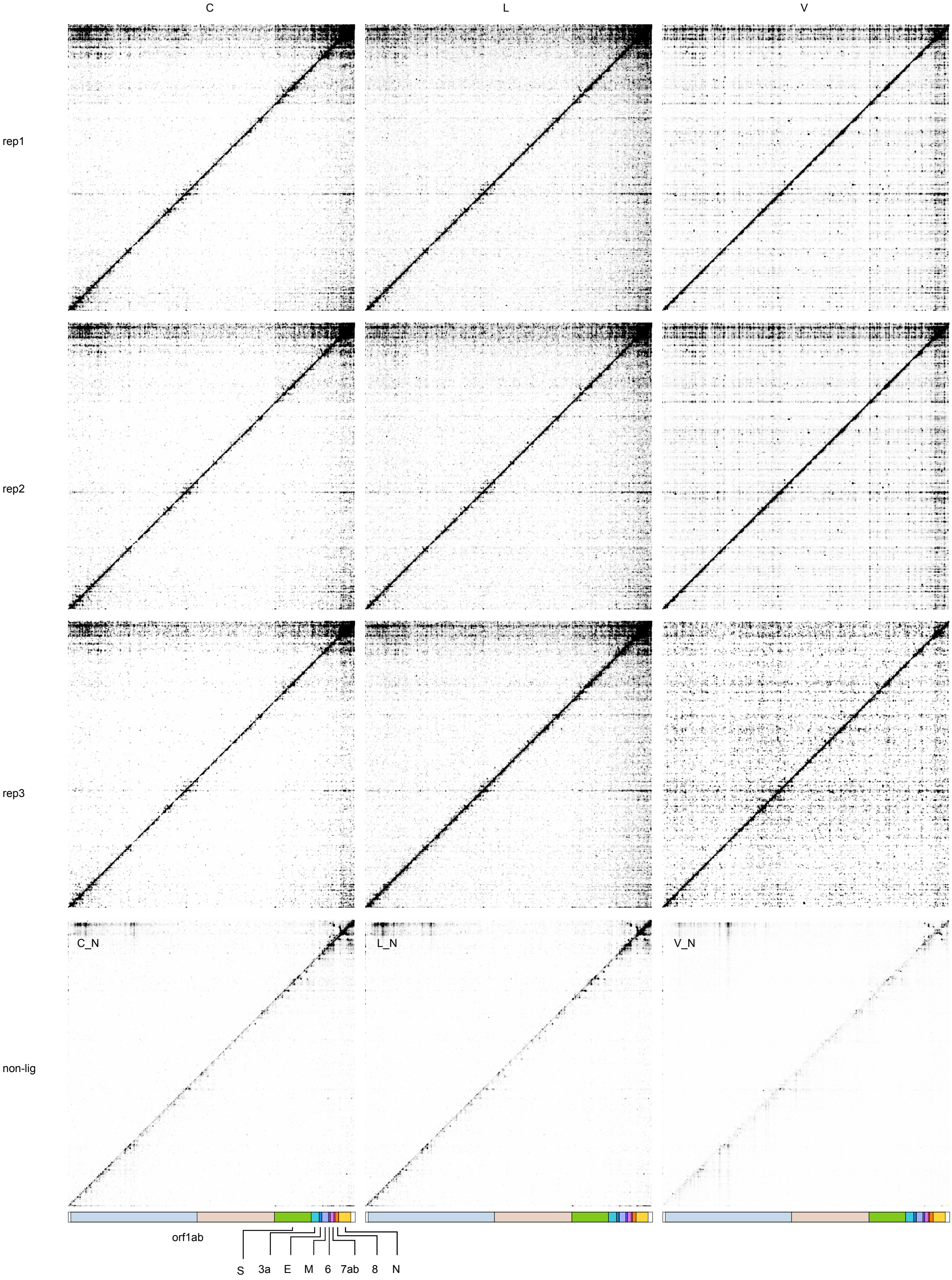

Supplementary Fig2

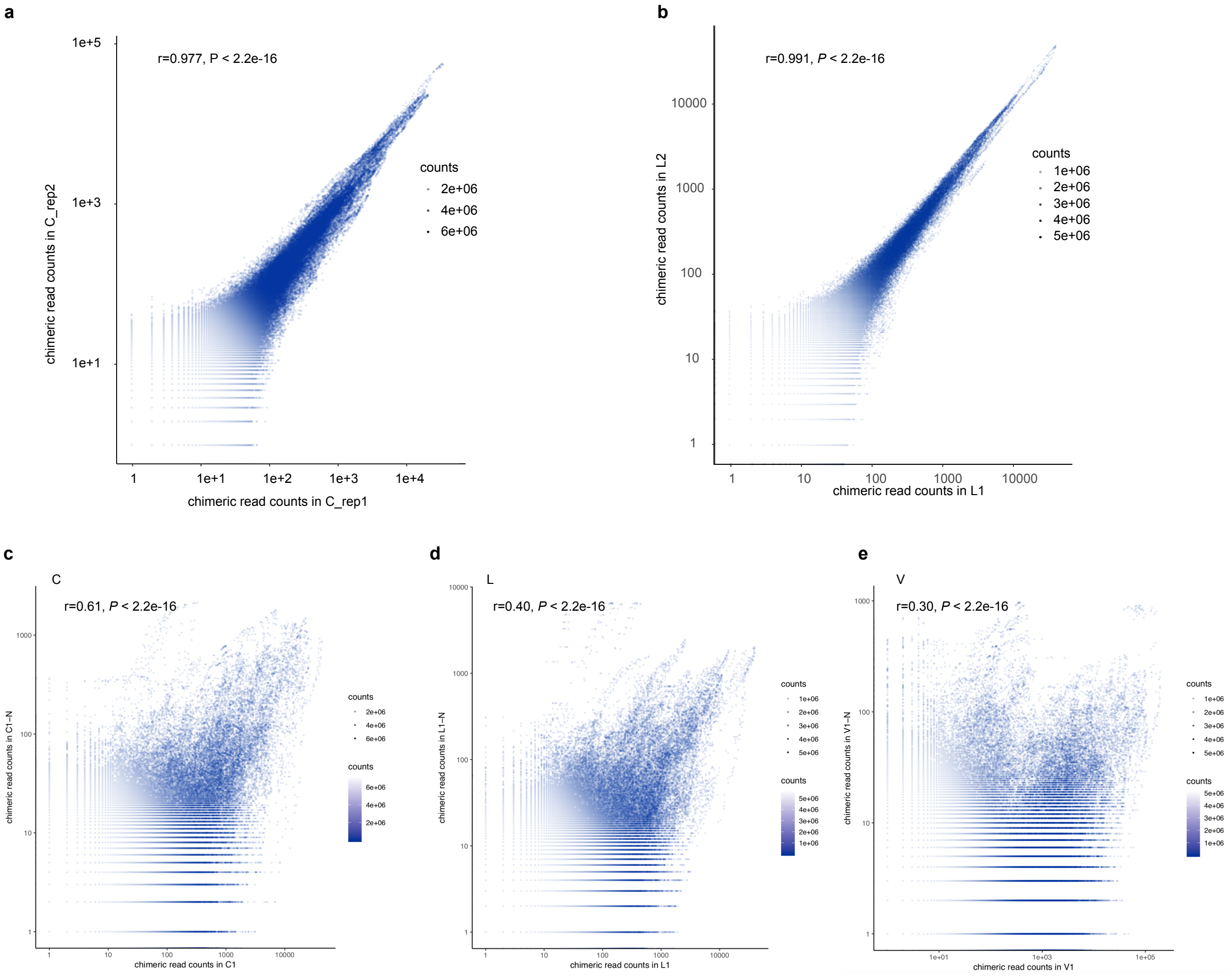

Supplementary Fig3

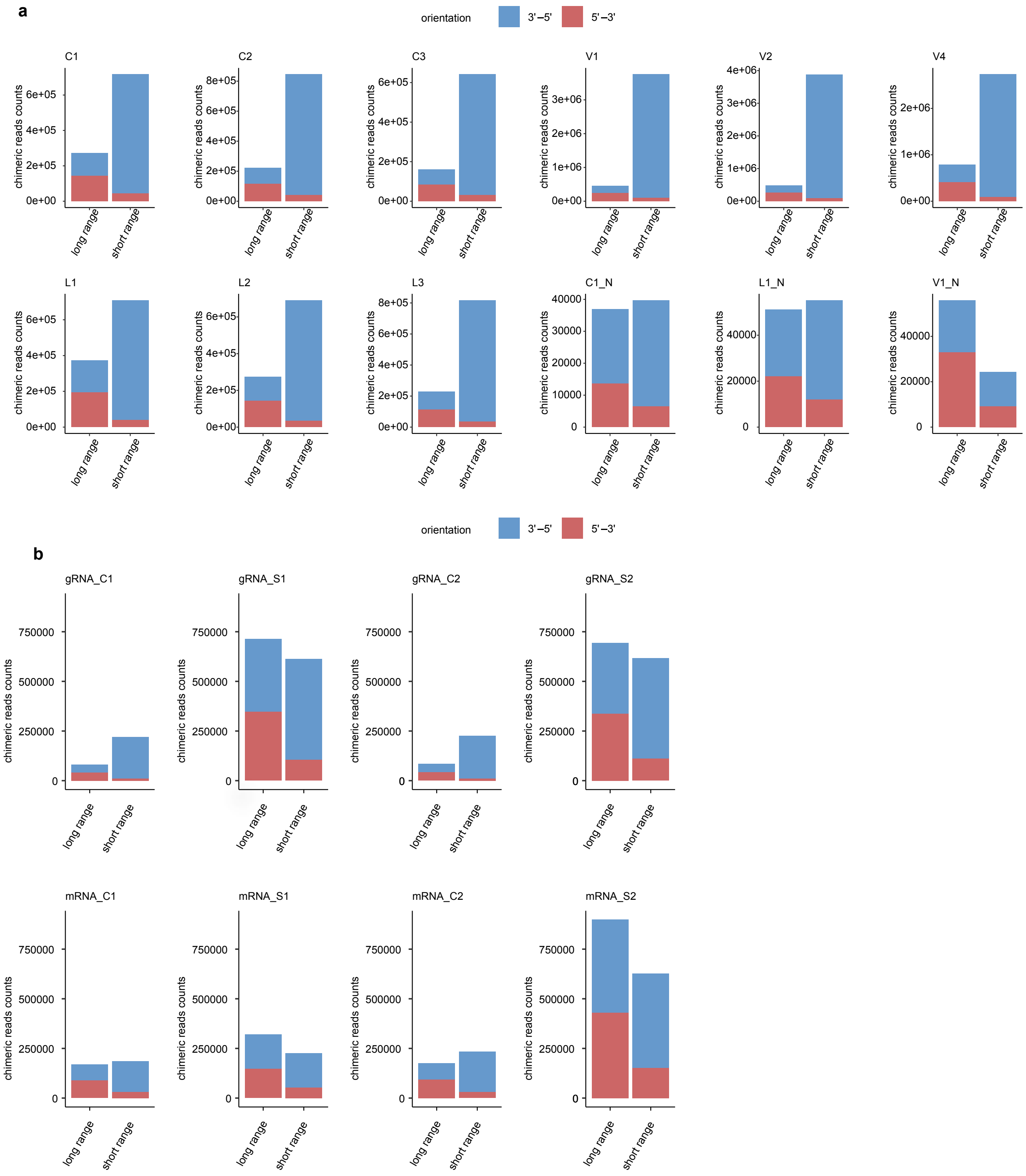

Supplementary Fig4

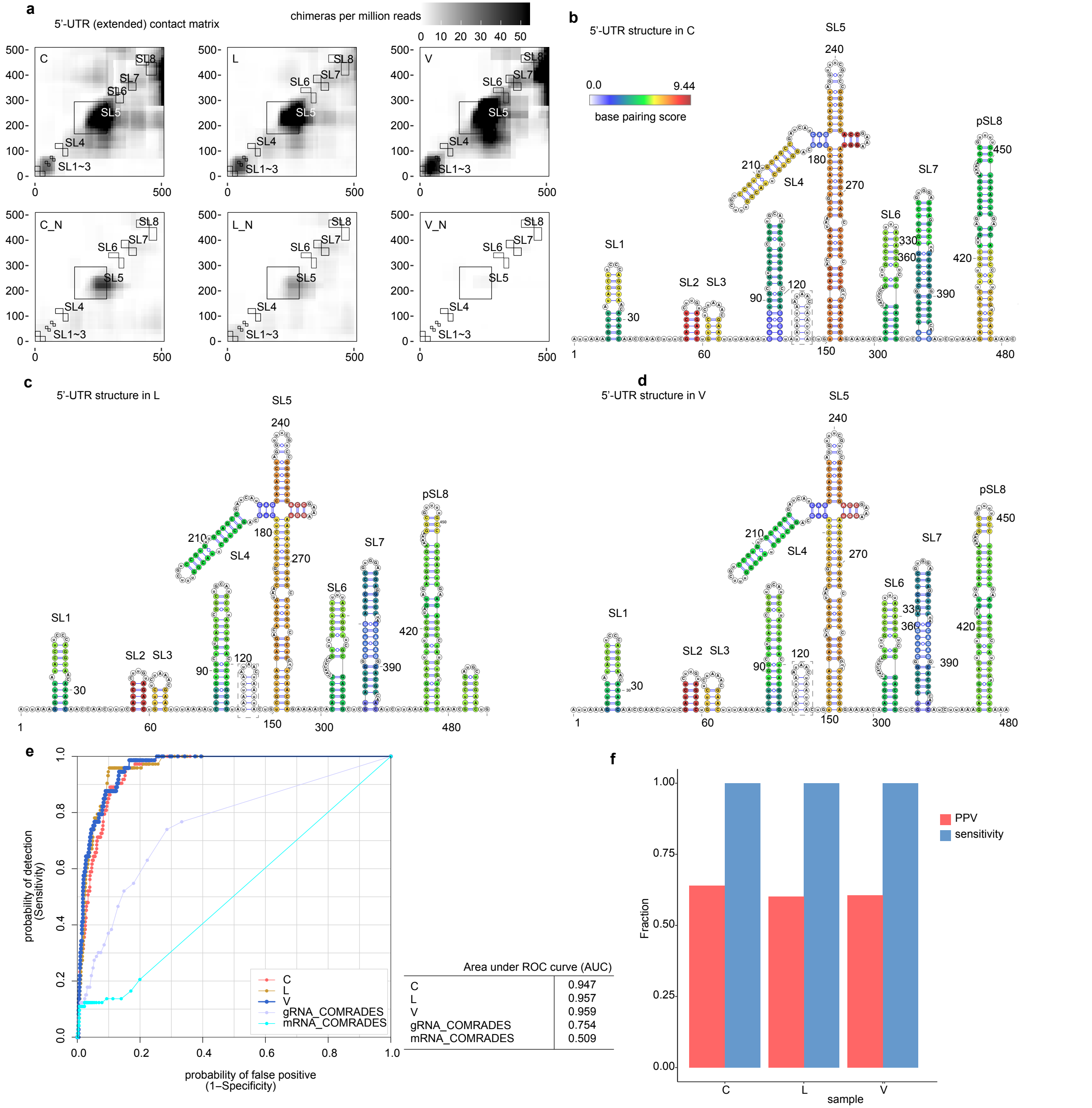

Supplementary Fig 5

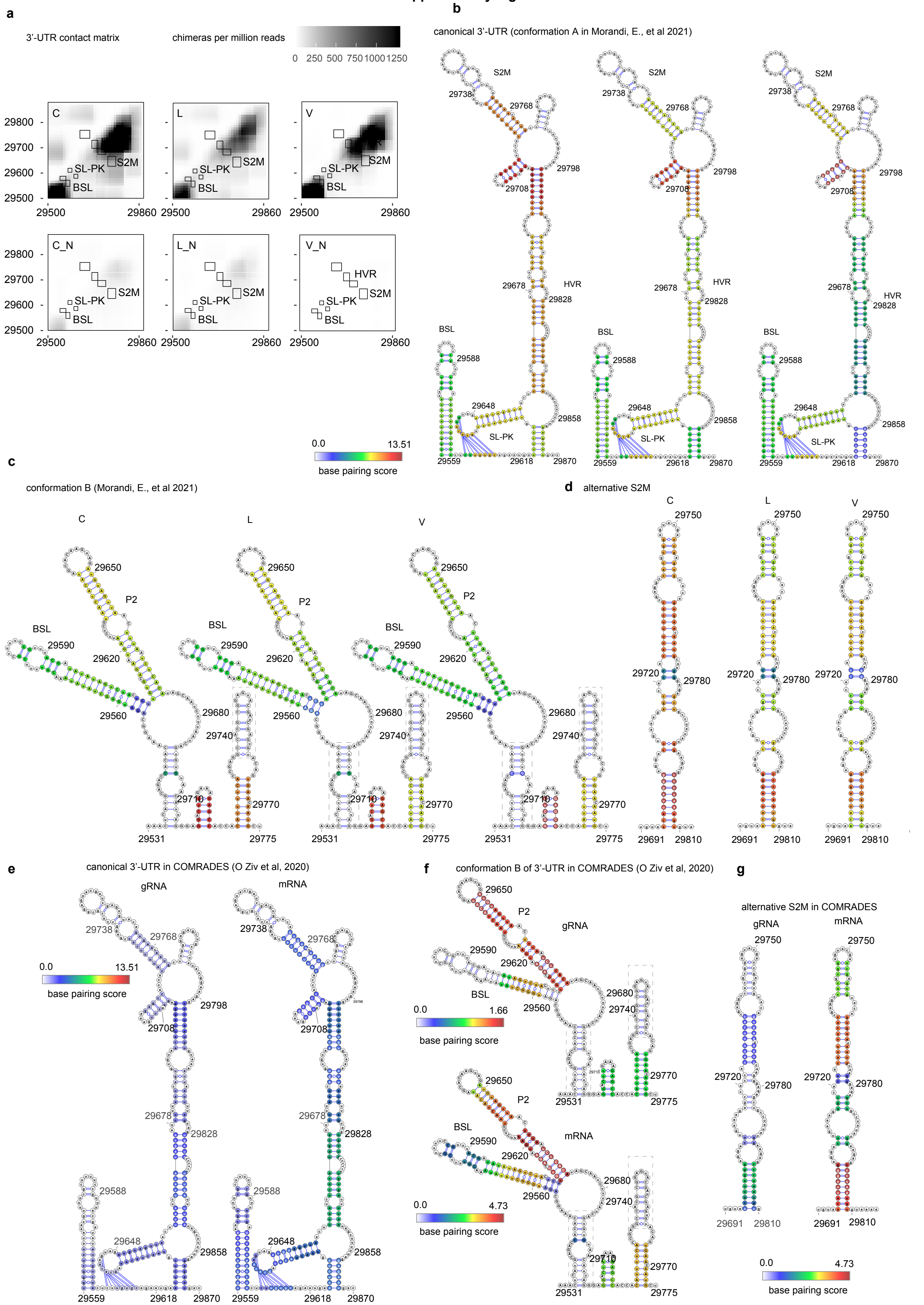

Supplementary Fig6

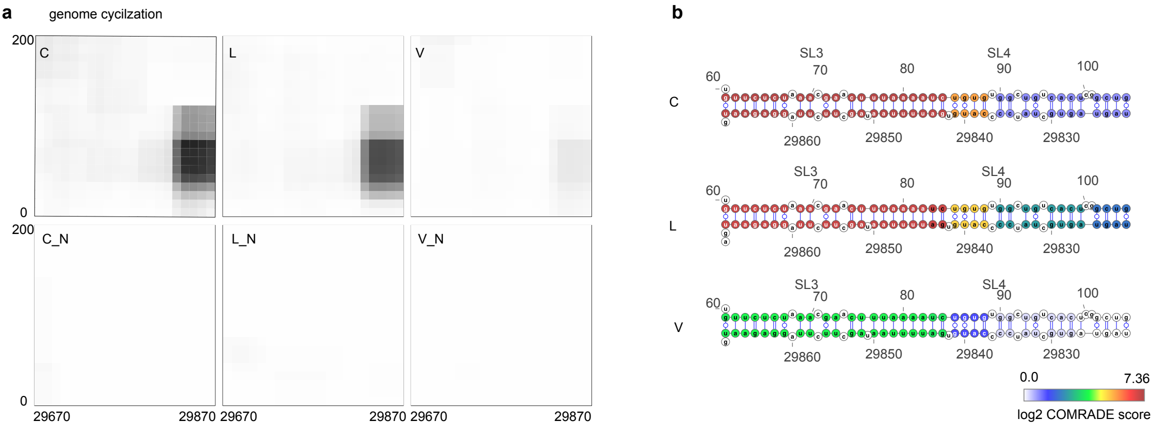

Supplementary Fig7

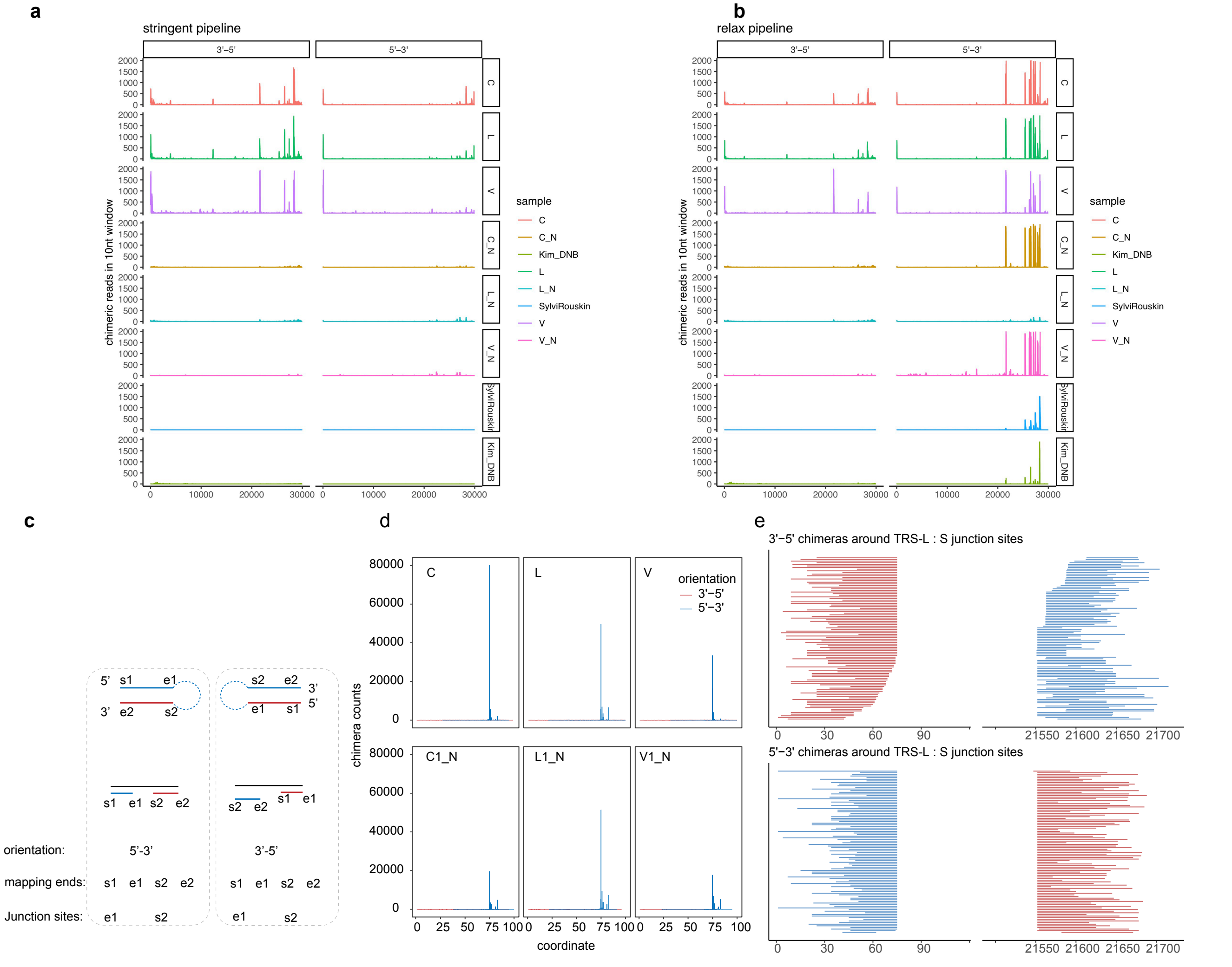

Supplementary Fig8

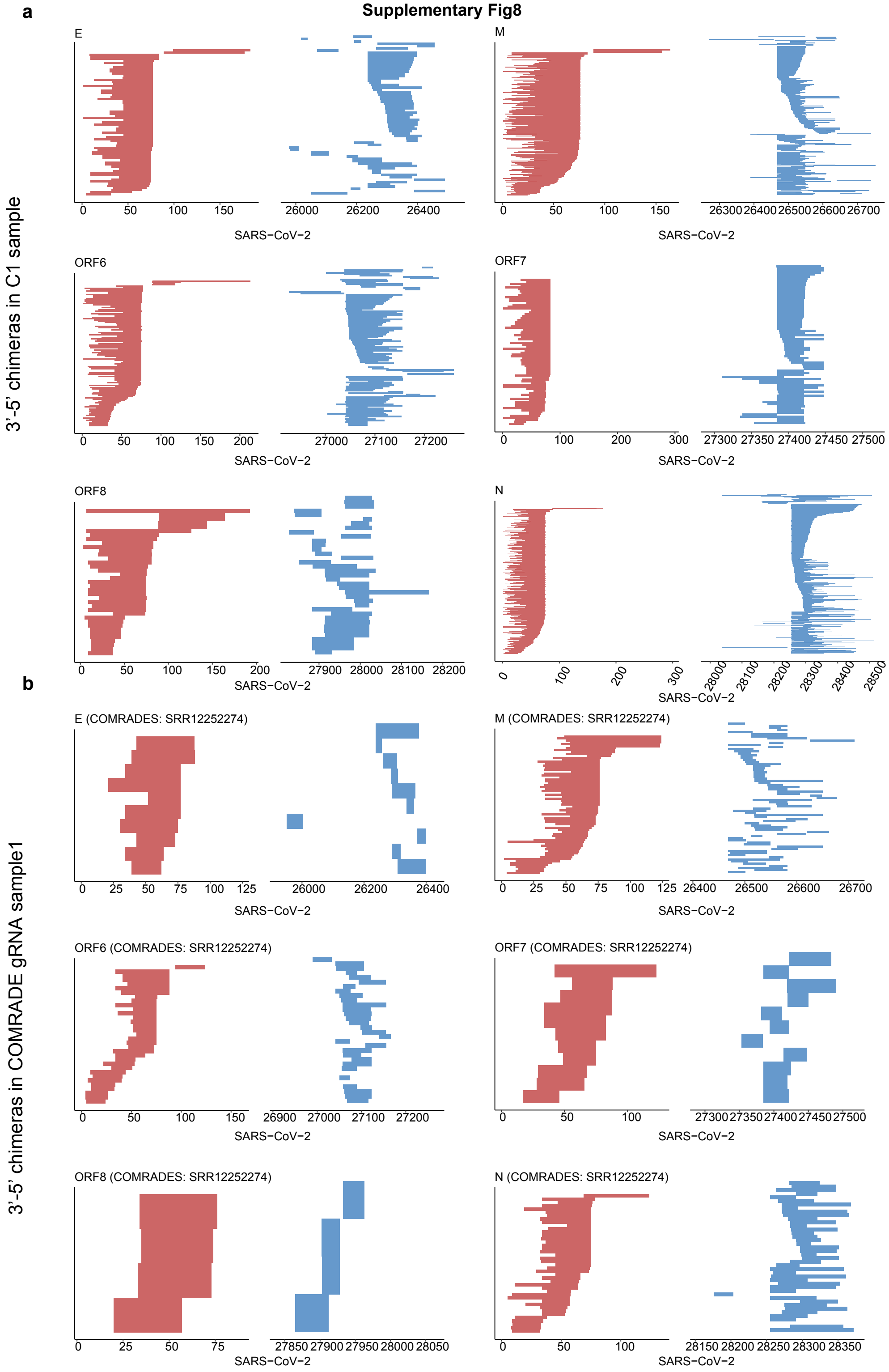

Supplementary Fig9

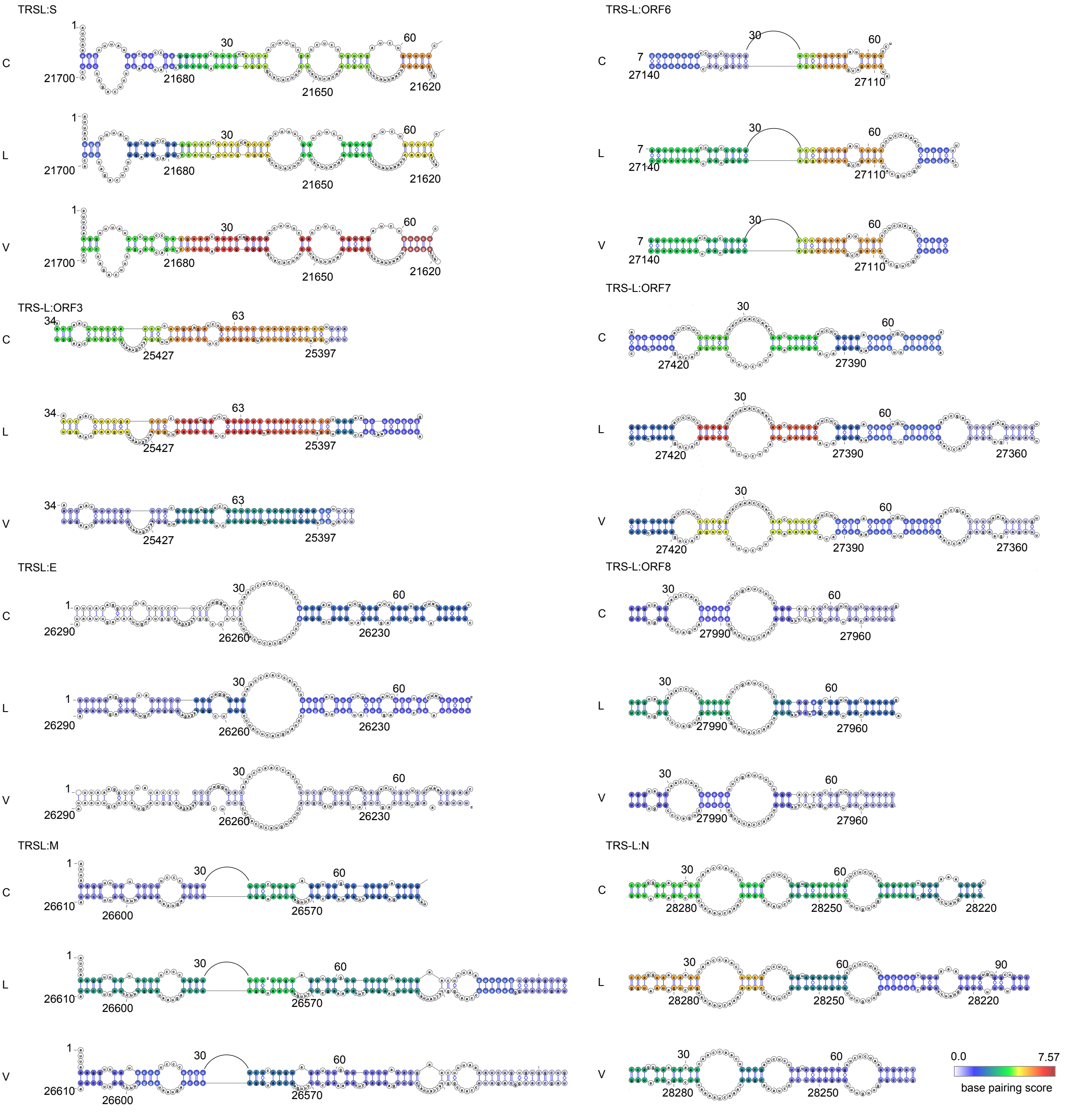

Supplementary Fig10

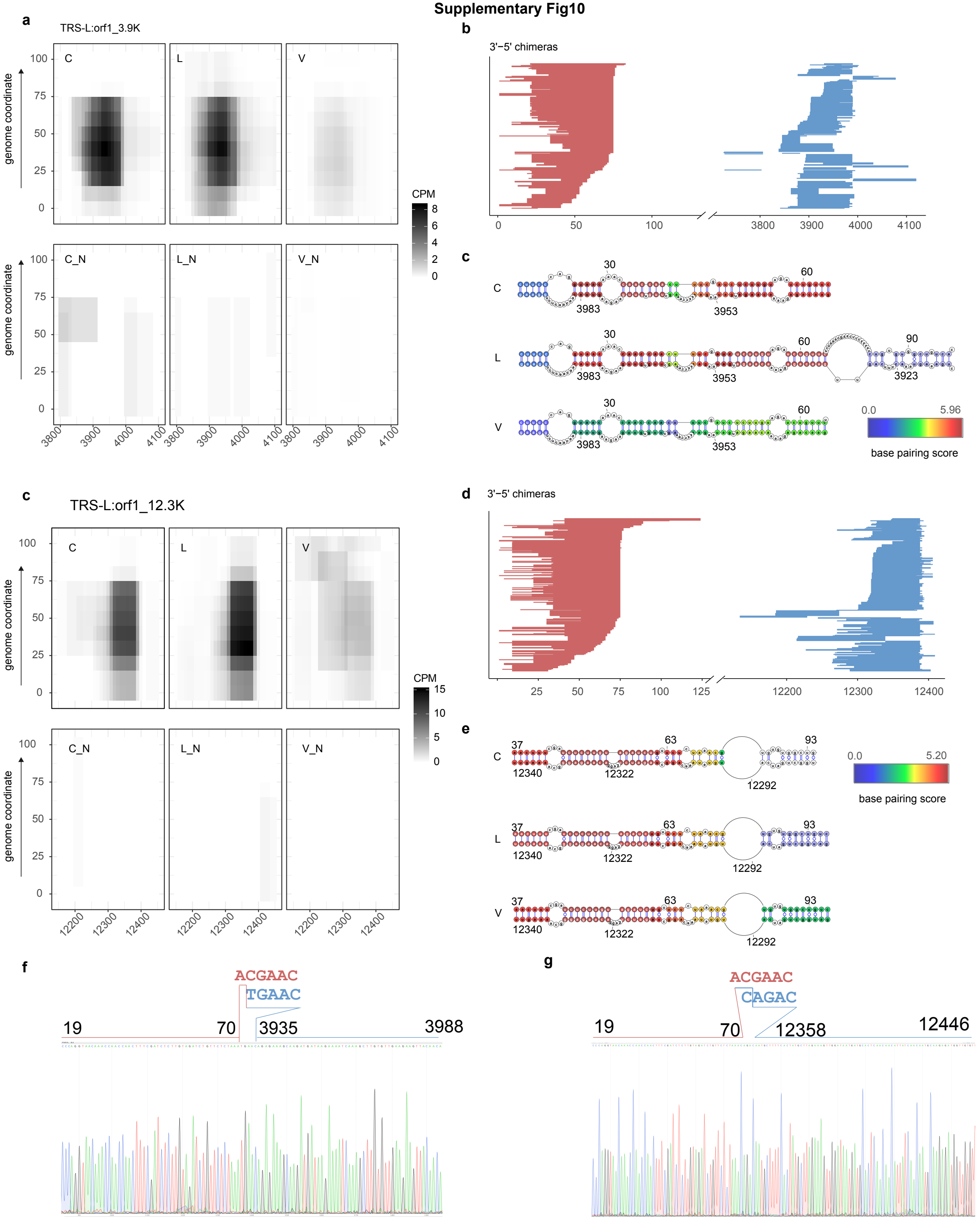

Supplementary Fig11

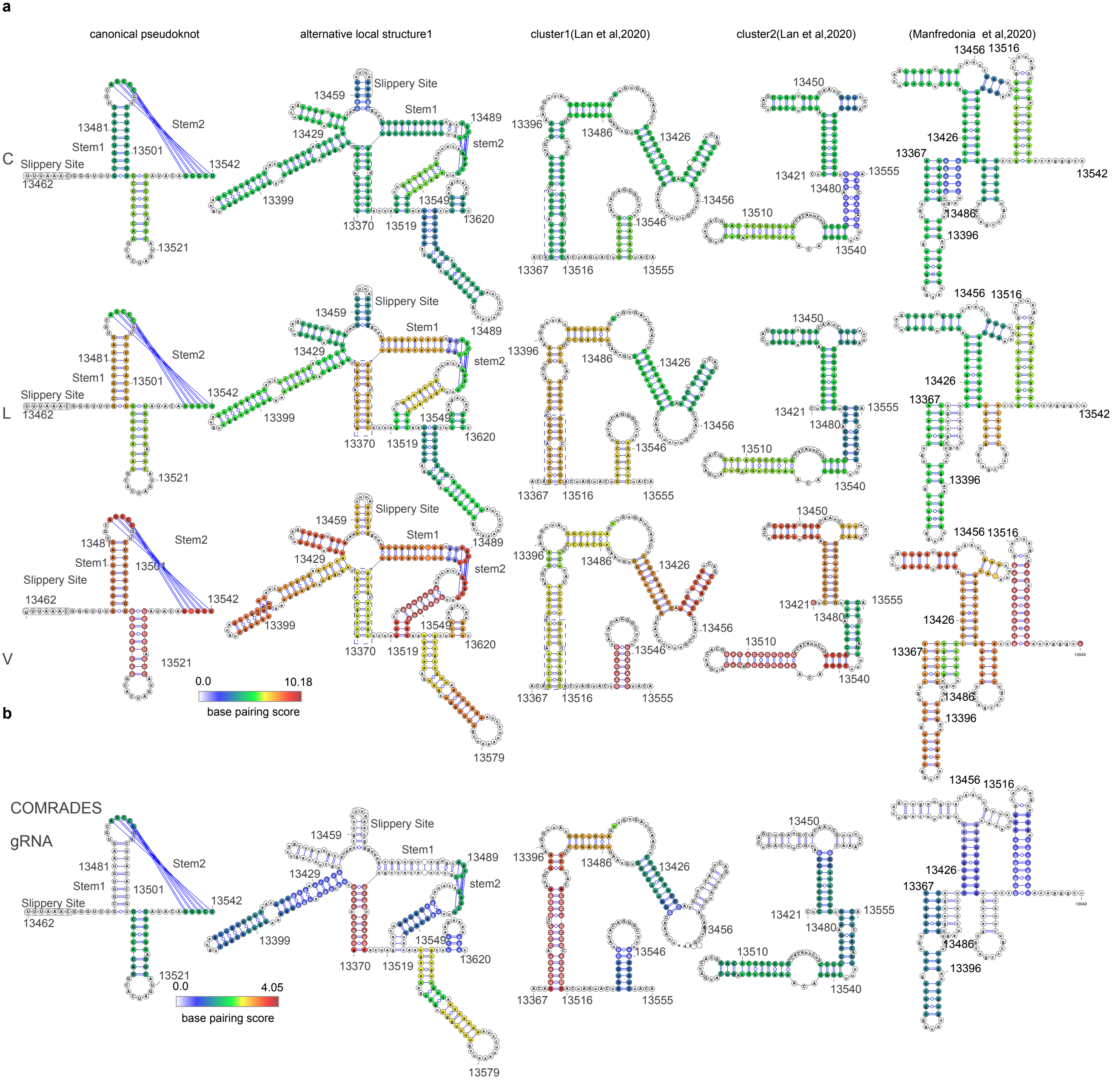

Supplementary Fig12

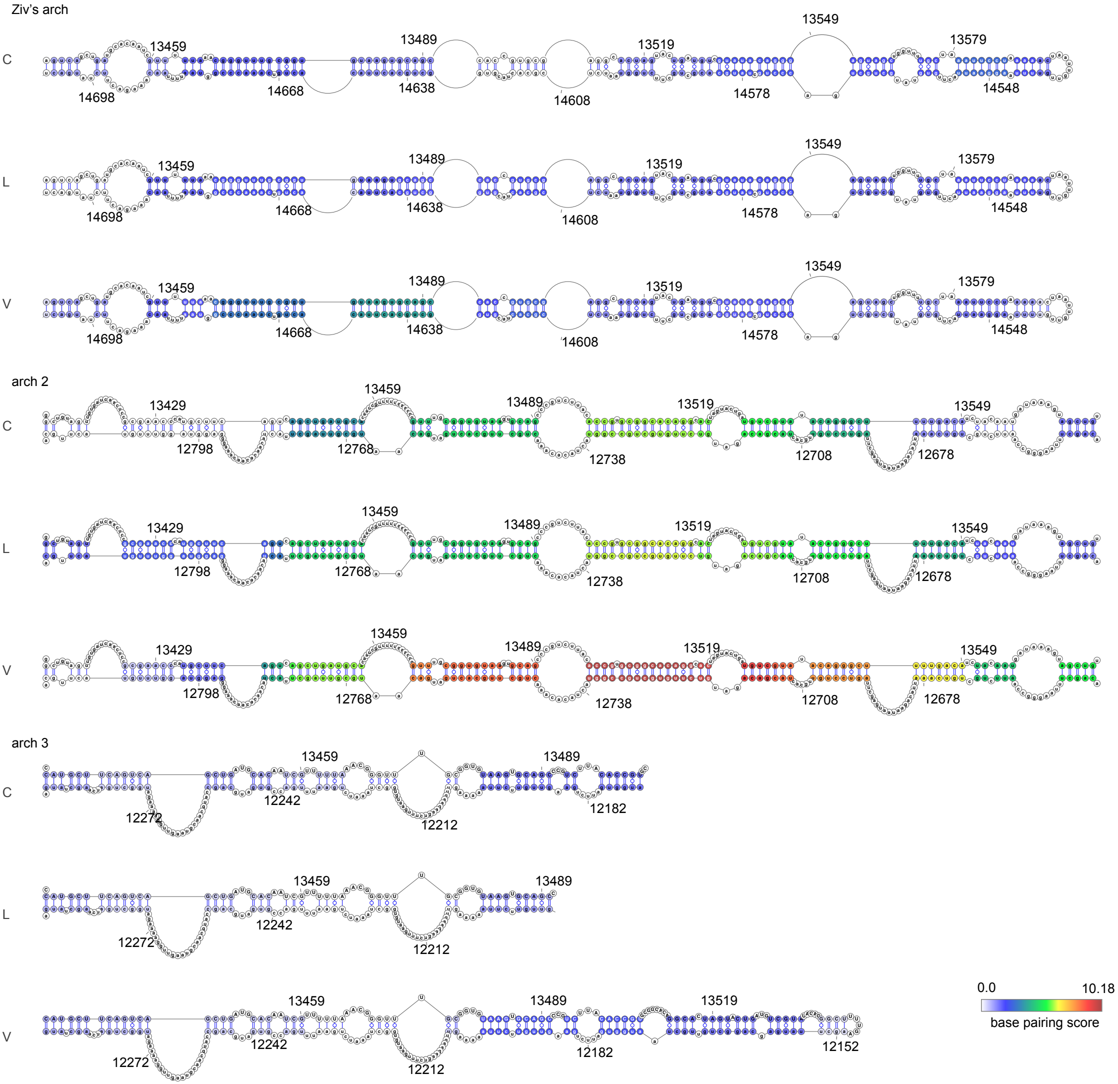

Supplementary Fig13

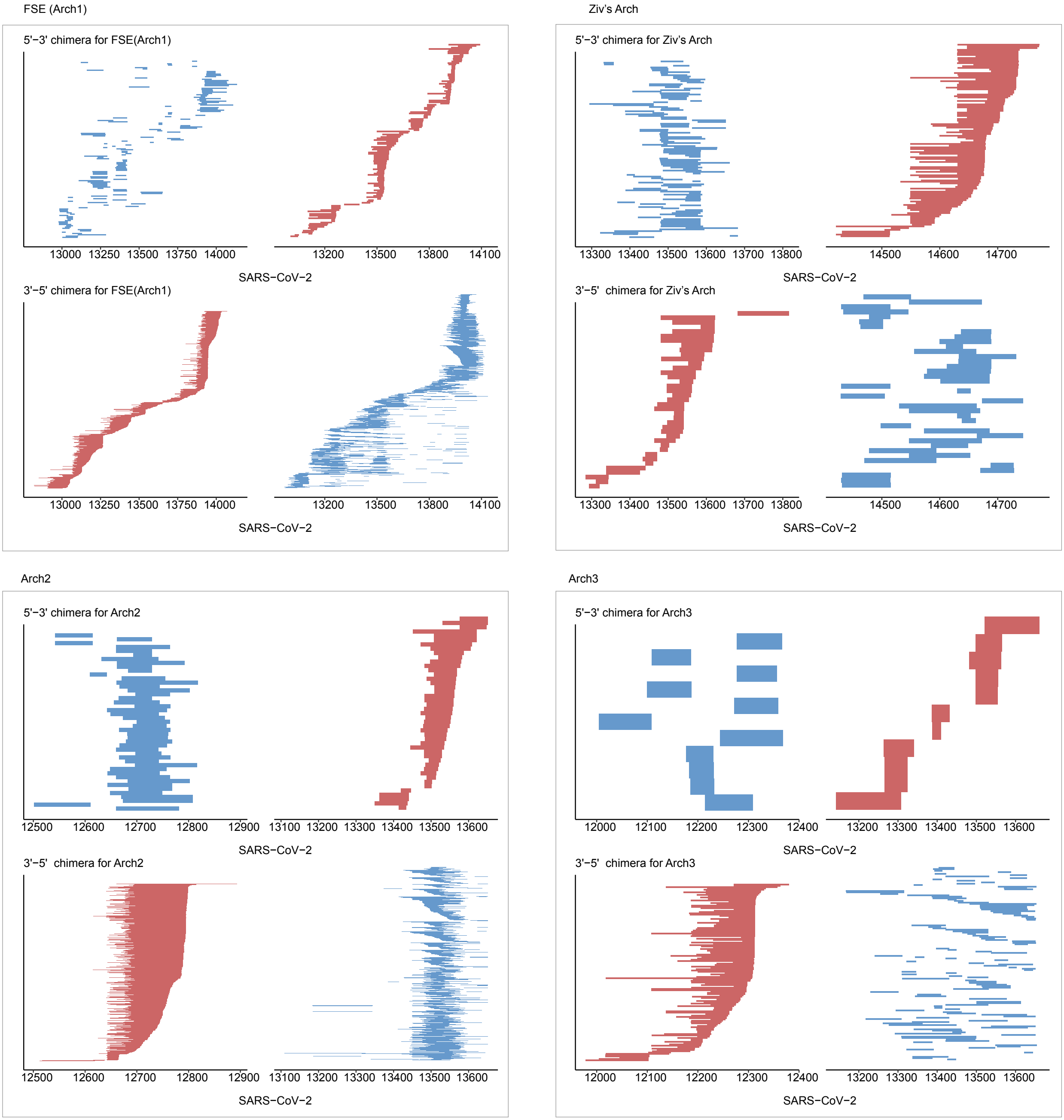

Supplementary Fig14

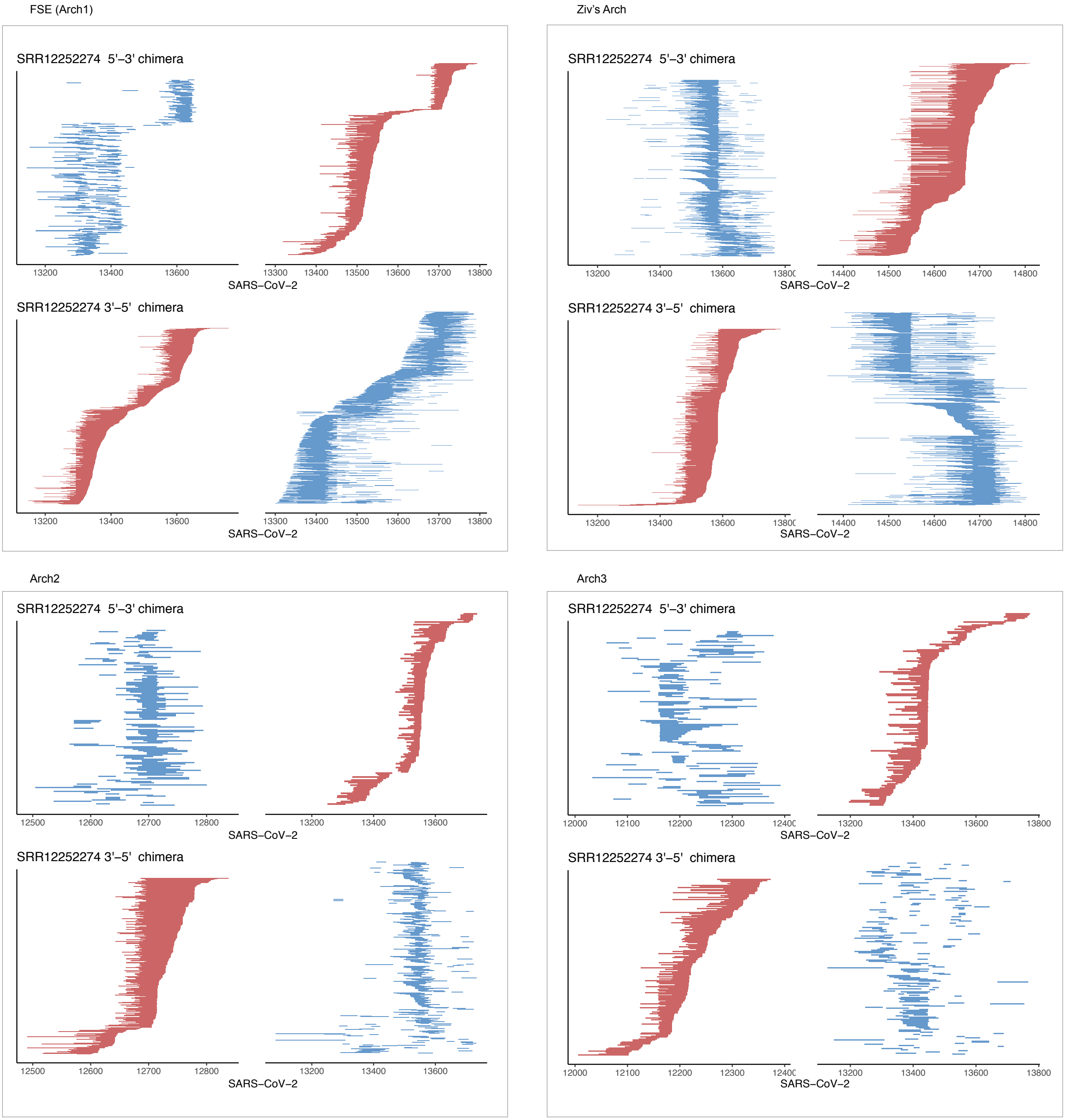

Supplementary Fig15

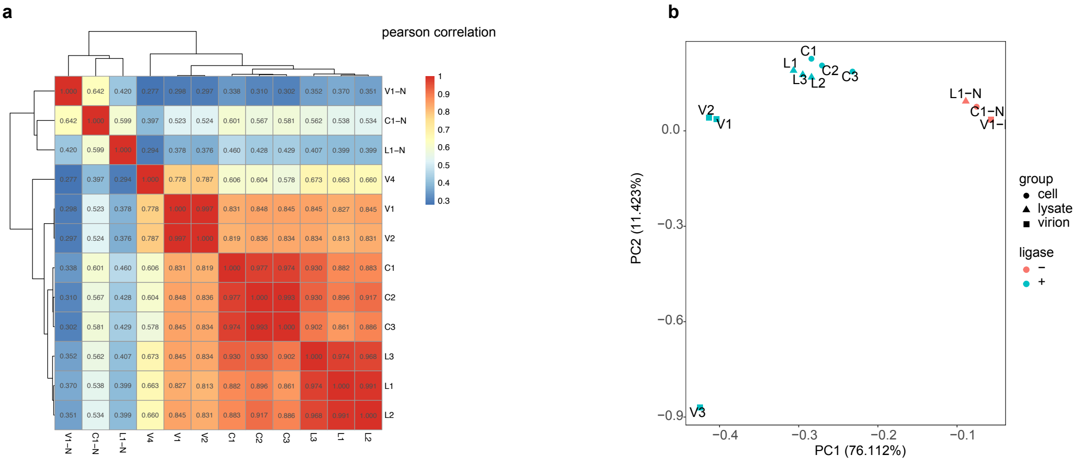

Supplementary Fig16

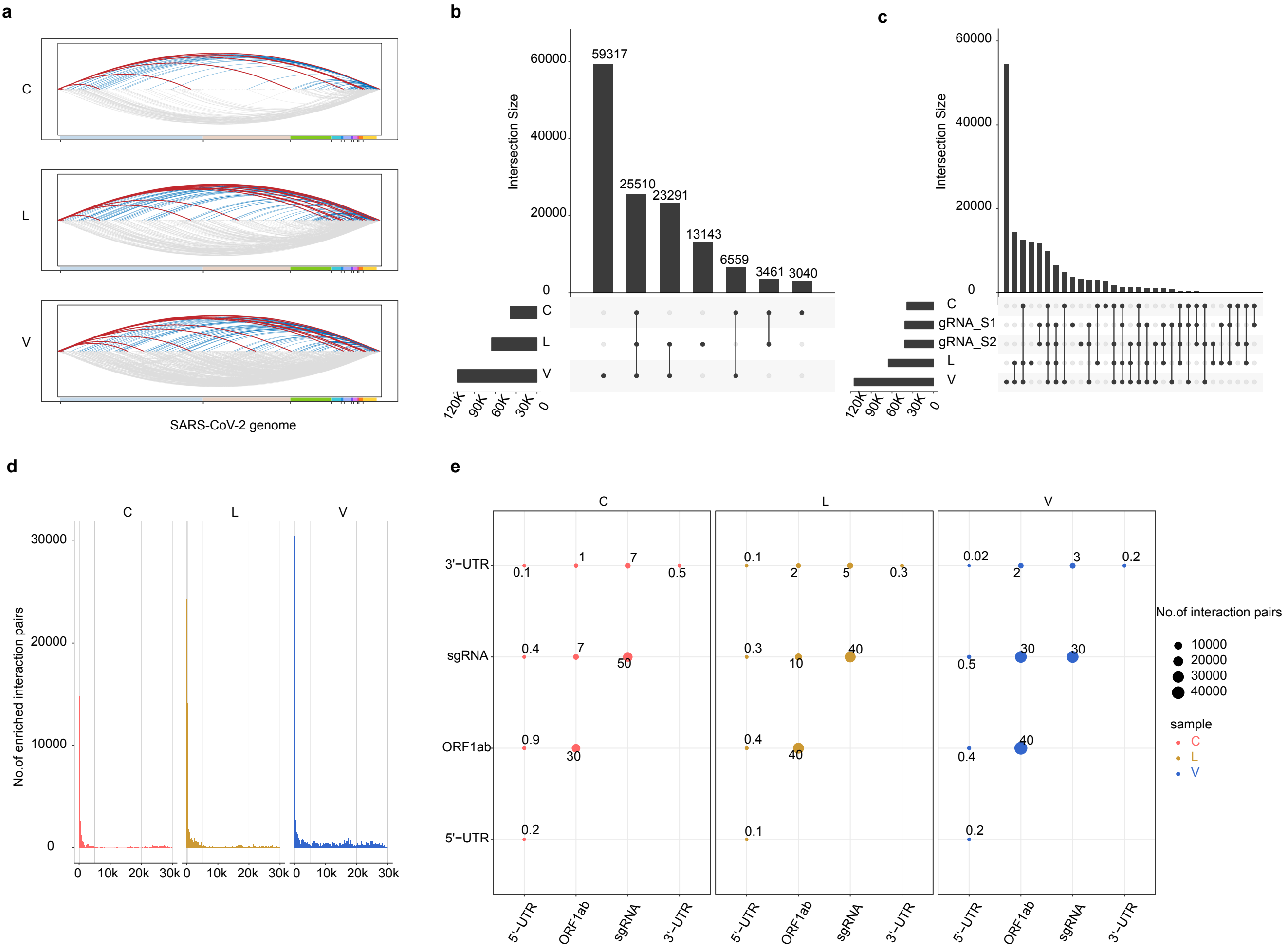

Supplementary Fig17

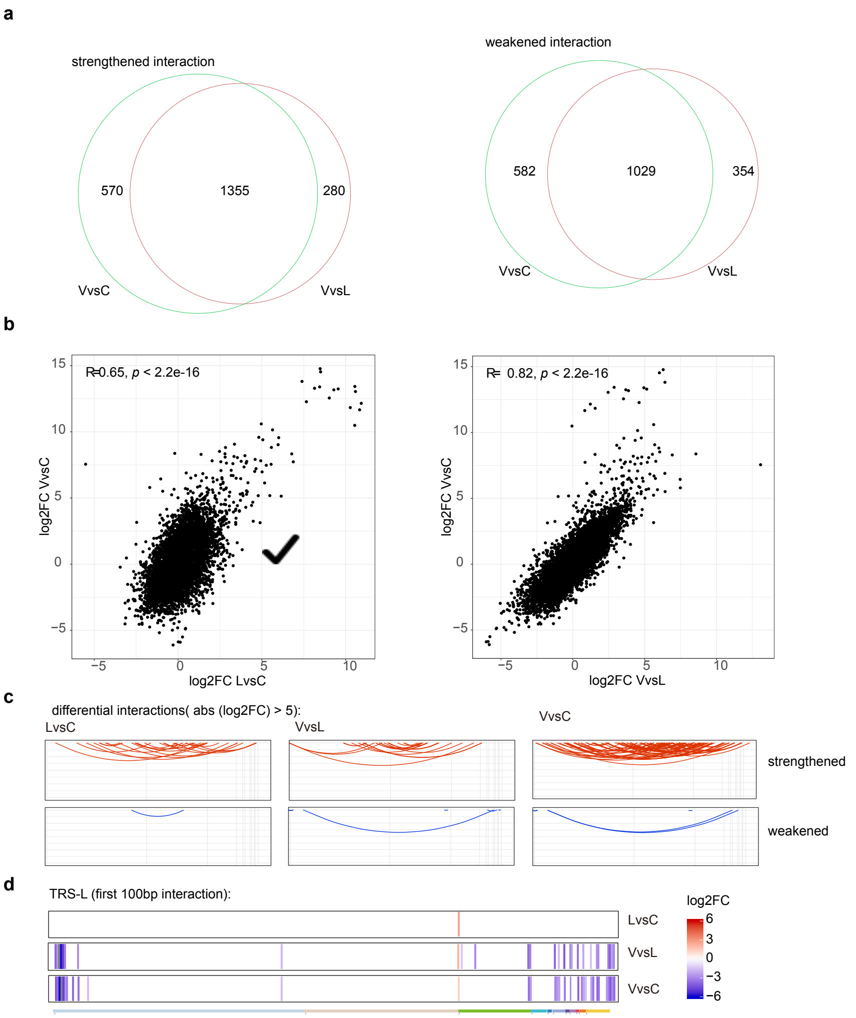

Supplementary Fig18

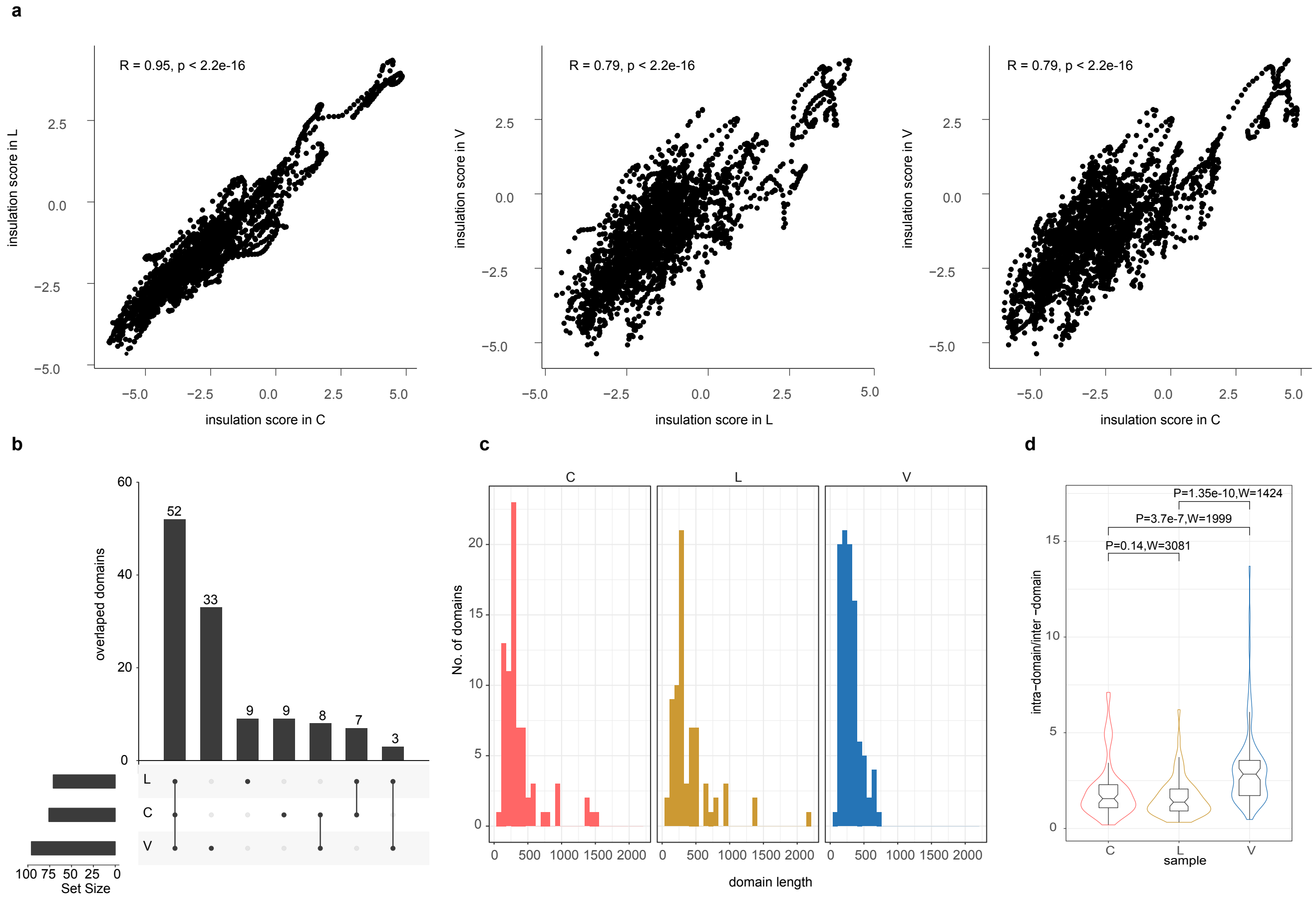

Supplementary Fig19

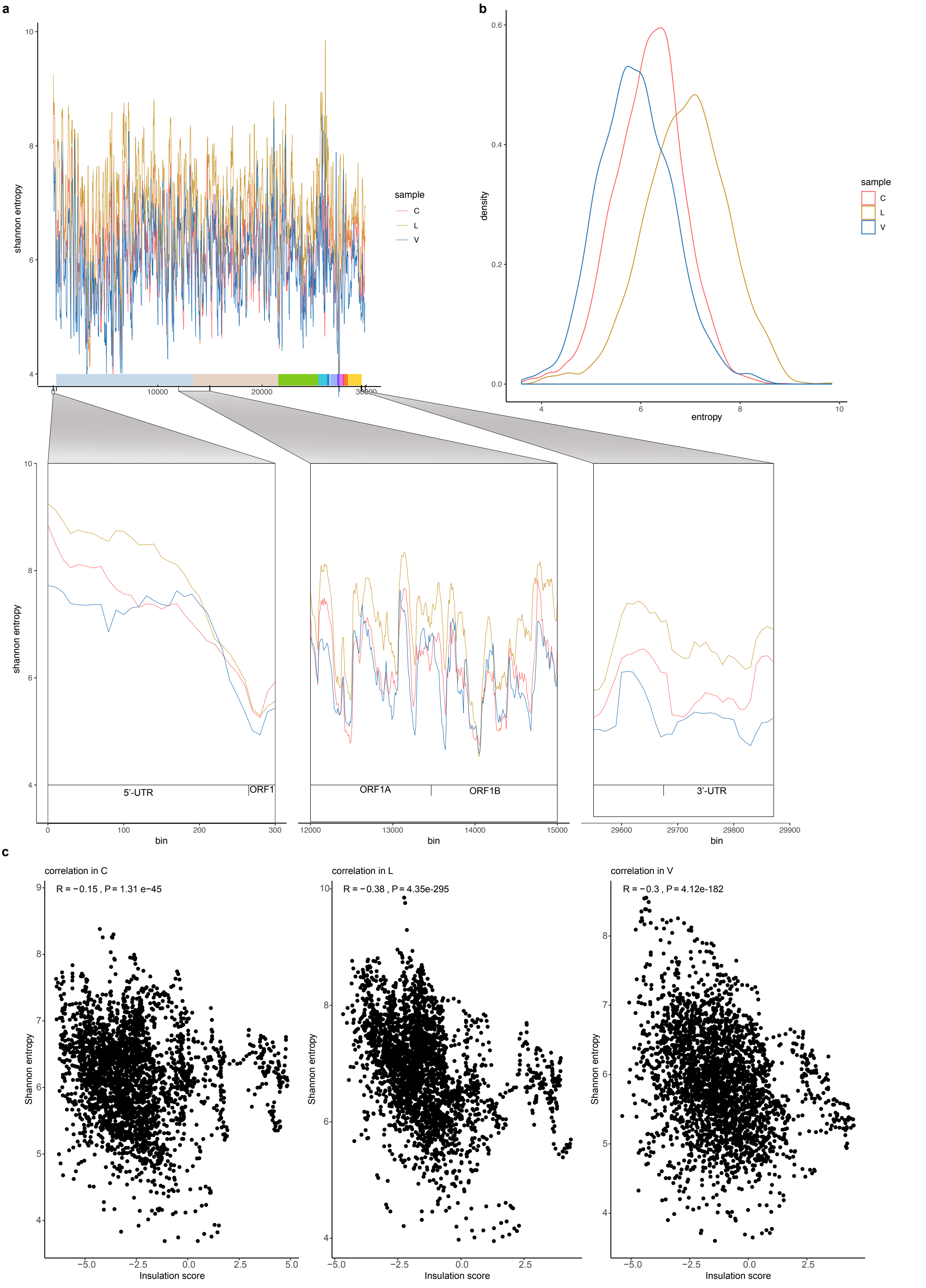

Supplementary Fig20

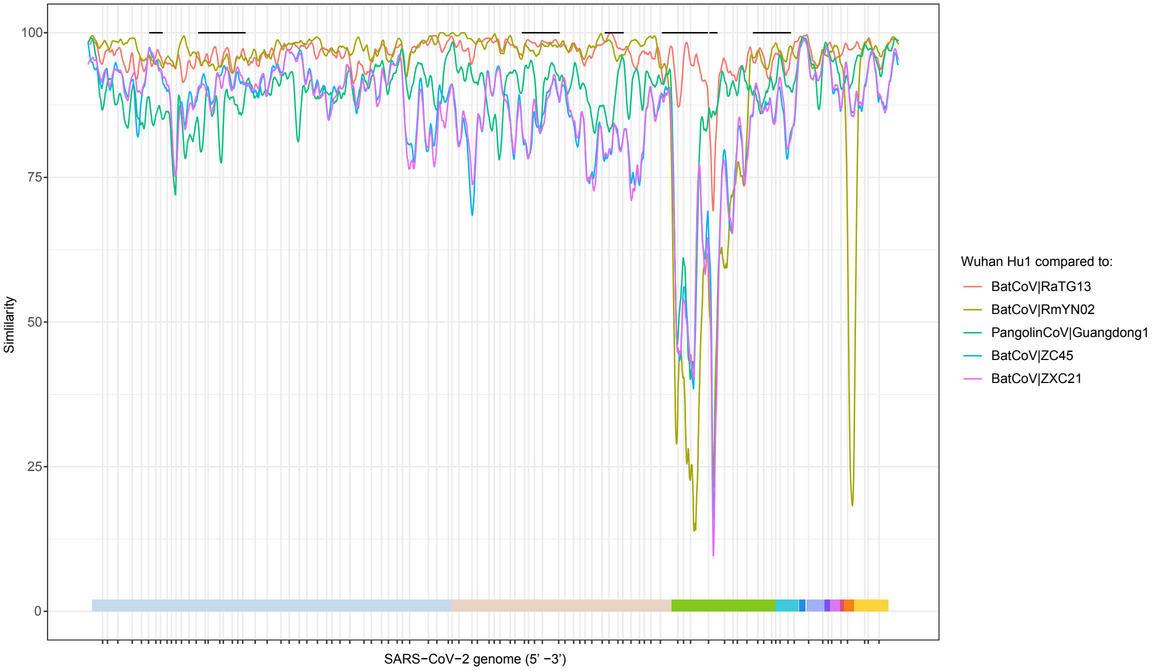

Supplement: Supplementary file 1 — Supplementary information [file 41467_2021_25999_MOESM1_ESM.pdf]
