# Supplementary material for: In vivo structure and dynamics of the SARS-CoV-2 RNA genome
Source: Nat Commun. 2021 Sep 28;12:5695. doi: 10.1038/s41467-021-25999-1 (PMC8478942; doi:10.1038/s41467-021-25999-1)
Supplement: Supplementary file 6 — Description of additional supplementary files [file 41467_2021_25999_MOESM6_ESM.docx]

Description of additional supplementary files

Title: Supplementary data1

Description: Statistics of mapping and chimeras of all the samples

Title: Supplementary data2

Description: contact matrix of each group of datasets.

Title: Supplementary data3

Description: DESeq2 statistics for enrichment of interaction bin pairs in ligated samples. The statistics of baseMean: mean of normalized counts for all samples; log2FoldChange: log2 fold change; lfcSE: standard error; stat: Wald statistic; pvalue: Wald test P values; padj: Benjamini & Hochberg adjusted P values.
