# Supplementary material for: In vivo structure and dynamics of the SARS-CoV-2 RNA genome
Source: Nat Commun. 2021 Sep 28;12:5695. doi: 10.1038/s41467-021-25999-1 (PMC8478942; doi:10.1038/s41467-021-25999-1)
Supplement: Supplementary file 7 — Reporting Summary [file 41467_2021_25999_MOESM7_ESM.pdf]

nature research

Corresponding author(s): Zhihu Zhao

Last updated by author(s): Aug 30, 2021

Reporting Summary

Nature Research wishes to improve the reproducibility of the work that we publish. This form provides structure for consistency and transparency in reporting. For further information on Nature Research policies, see our Editorial Policies and the Editorial Policy Checklist.

Statistics

For all statistical analyses, confirm that the following items are present in the figure legend, table legend, main text, or Methods section.

n/a	Confirmed	
☐	☒	The exact sample size (*n*) for each experimental group/condition, given as a discrete number and unit of measurement
☐	☒	A statement on whether measurements were taken from distinct samples or whether the same sample was measured repeatedly
☐	☒	The statistical test(s) used AND whether they are one- or two-sided<br>*Only common tests should be described solely by name; describe more complex techniques in the Methods section.*
☒	☐	A description of all covariates tested
☐	☒	A description of any assumptions or corrections, such as tests of normality and adjustment for multiple comparisons
☐	☒	A full description of the statistical parameters including central tendency (e.g. means) or other basic estimates (e.g. regression coefficient) AND variation (e.g. standard deviation) or associated estimates of uncertainty (e.g. confidence intervals)
☐	☒	For null hypothesis testing, the test statistic (e.g. *F*, *t*, *r*) with confidence intervals, effect sizes, degrees of freedom and *P* value noted<br>*Give P values as exact values whenever suitable.*
☒	☐	For Bayesian analysis, information on the choice of priors and Markov chain Monte Carlo settings
☒	☐	For hierarchical and complex designs, identification of the appropriate level for tests and full reporting of outcomes
☐	☒	Estimates of effect sizes (e.g. Cohen's *d*, Pearson's *r*), indicating how they were calculated

Our web collection on statistics for biologists contains articles on many of the points above.

Software and code

Policy information about availability of computer code

Data collection	sequencing Data was collected by Illumina CASAVA 1.8
Data analysis	Microsoft Excel 2017, Python 3.6, R 3.5.3, numpy 1.15.4, scipy 1.3.0,pandas 0.23.4, dplyr 0.8.0.1, DeSeq2 1.22.2, ggplot2 3.1.0 , Java Treeview 1.1.6r4, VARNA v3-93, caTools 1.17 ,Hyb (https://github.com/gkudla/hyb),COMRADES (https://github.com/gkudla/comrades),. Detailed methods are described in methods, and custom codes were deposited to Github (https://github.com/zany1983/simplifed_SPLASH)

For manuscripts utilizing custom algorithms or software that are central to the research but not yet described in published literature, software must be made available to editors and reviewers. We strongly encourage code deposition in a community repository (e.g. GitHub). See the Nature Research guidelines for submitting code & software for further information.

Data

Policy information about availability of data

All manuscripts must include a data availability statement. This statement should provide the following information, where applicable:

- Accession codes, unique identifiers, or web links for publicly available datasets
- A list of figures that have associated raw data
- A description of any restrictions on data availability

The simplified SPLASH data generated in this study, along with Processed sequencing datasets analyzed in this study (hyb files) have been deposited in the Gene Expression Omnibus (GEO) database under accession code GSE164565 [https://www.ncbi.nlm.nih.gov/geo/query/acc.cgi?acc=GSE164565]. DESeq2 statistics for enrichment of interaction bin pairs in ligated samples are provided in supplementary data3. The COMRADES data used in this study are available in the GEO database under accession GSE154662 [https://www.ncbi.nlm.nih.gov/geo/query/acc.cgi?acc=GSE154662]. The SARS-COV-2 refference genome can be cound at https://www.ncbi.nlm.nih.gov/genome/?term=NC_045512.2.

Field-specific reporting

Please select the one below that is the best fit for your research. If you are not sure, read the appropriate sections before making your selection.

☒ Life sciences ☐ Behavioural & social sciences ☐ Ecological, evolutionary & environmental sciences

For a reference copy of the document with all sections, see nature.com/documents/nr-reporting-summary-flat.pdf

Life sciences study design

All studies must disclose on these points even when the disclosure is negative.

Sample size	No sample size calculations were performed. Cells from 3 independent replicates were analysed for reproducibility. This sample size was chosen to match mostly published work . Due to the low variability between these vitro experiments, this sample size is also widely accepted in the field, for example COMRADES (Ziv O, et al. Mol Cell. 2020) and SPLASH (Aw JG, et al. Mol Cell. 2016;62(4):603-17.)
Data exclusions	No data were excluded from analyses.
Replication	Whenever possible, readouts were performed with 3 independent replicates. All attempts at replication were successful.
Randomization	Groups were determined by different time of culture, therefore, randomization was not applicable.
Blinding	Investigators who perform simplified SPLASH experiment and high throughput sequencing data collection and analysis was performed blindly and automatically. For other experiments,cell lines were picked and treated by the two individuals.

Reporting for specific materials, systems and methods

We require information from authors about some types of materials, experimental systems and methods used in many studies. Here, indicate whether each material, system or method listed is relevant to your study. If you are not sure if a list item applies to your research, read the appropriate section before selecting a response.

Materials & experimental systems

n/a	Involved in the study	
☒	☐	Antibodies
☐	☒	Eukaryotic cell lines
☒	☐	Palaeontology and archaeology
☒	☐	Animals and other organisms
☒	☐	Human research participants
☒	☐	Clinical data
☒	☐	Dual use research of concern

Methods

n/a	Involved in the study	
☒	☐	ChIP-seq
☒	☐	Flow cytometry
☒	☐	MRI-based neuroimaging

Eukaryotic cell lines

Policy information about cell lines

Cell line source(s)	Chlorocebus sabaeus (Green monkey) VeroE6 (female, RRID:CVCL_YQ49) were purchased from American Type Culture Collection (ATCC, id: ATCC CRL-1586).
Authentication	Cells not authenticated
Mycoplasma contamination	Cells negative for mycoplasma.
Commonly misidentified lines (See ICLAC register)	Not used
